# P091 Giant cell arthritis with tongue necrosis in a patient with limited scleroderma a diagnostic challenge

**DOI:** 10.1093/rap/rkag113.121

**Published:** 2026-11-03

**Authors:** Fatima Salih, James Beynon, Ukachukwu Okoro, Ziad Chilmeran

**Affiliations:** Rheumatology Department, University Hospital Southampton Foundation Trust, Southampton, United Kingdom; Rheumatology Department, University Hospital Southampton Foundation Trust, Southampton, United Kingdom; Rheumatology Department, University Hospital Southampton Foundation Trust, Southampton, United Kingdom; Rheumatology Department, University Hospital Southampton Foundation Trust, Southampton, United Kingdom

## Abstract

**Introduction:**

Giant cell arteritis (GCA) is the most common medium- to large-vessel vasculitis in the UK, affecting 1 to 2 people per 10,000. It usually presents with headache, scalp tenderness, visual symptoms, and jaw claudication; it can rarely present with tongue necrosis in around 1% of all GCA cases.

Tongue necrosis is a rare clinical finding because of its rich collateral circulation, and it is usually unilateral, with GCA being the most common cause.

Limited scleroderma is the commonest form of scleroderma, causing calcinosis, Raynaud’s phenomenon, esophageal dysmotility, sclerodactyly and telangiectasia.

Raynaud’s phenomenon can rarely affect the tongue, causing lingual Raynaud’s.

**Case description:**

A 76-year-old lady with a past medical history of hypertension, ischemic heart disease (IHD), limited scleroderma and right big toe amputation was on lansoprazole 30, ramipril 5, amlodipine 5 mg, aspirin 75 mg, and atorvastatin 80 mg.

She was diagnosed with limited scleroderma in 2003. Her main manifestations were hand Raynaud’s phenomenon, started at the age of 20, puffy fingers, telangiectasia, and gastro-oesophageal reflux disease (GORD). Her systemic sclerosis symptoms have remained relatively stable over the years.

She has a significant coronary artery disease, with two coronary artery stentings in 2001, and in 2012, with a strong family history of IHD

In 2008, she developed digital ischaemia affecting the right great toe and the left second and third toes. Leading to auto-amputated. She received intravenous iloprost during this episode but was unable to tolerate treatment. She also underwent hyperbaric oxygen therapy, which improved her Raynaud’s symptoms.

In May, she presented with a two-month history of oral pain and right-sided jaw pain, inability to speak or eat, and worsening headache with tenderness around the ear. She denied other symptoms.

She was initially assessed by a dentist and underwent the extraction of several teeth. Her condition progressed to severe tongue ischemia. The tongue became necrotic and black. So she was admitted for surgical debridement and intravenous antibiotics due to concern regarding superimposed tongue infection/abscess and intravenous methylprednisolone.

Subsequent investigation showed a positive temporal artery ultrasound with a negative PET scan, non-conclusive temporal artery biopsy, and aortic arch CT Angio showing atherosclerotic disease in the internal carotids.

During her admission, she had multiple tongue debridement, and her headache and jaw symptoms improved significantly with the high-dose steroid, but because of the swallowing difficulty, she was offered enteral feeding, but she refused and had palliative care.

**Discussion:**

Tongue necrosis is a well-known complication of GCA, but a review of the literature found few case reports of tongue necrosis in carotid artery stenosis. This made the diagnosis more challenging. The diagnosis was challenging in this case due to the overlap between features of GCA, severe systemic sclerosis-related vasculopathy, and previous episodes of tongue Raynaud’s phenomenon.

Initially, the patient did not report significant scalp tenderness and later provided a history suggestive of headache, which made the clinical picture less straightforward. This was further complicated by her extensive background of vasculopathy, including severe Raynaud’s phenomenon, previous digital ischemia with auto-amputation, known coronary artery disease with previous myocardial infarctions and stenting, and a previous history of tongue Raynaud’s phenomenon.

Given her underlying limited cutaneous systemic sclerosis, there was also concern regarding the risks of administering high-dose corticosteroids, particularly the potential to precipitate scleroderma renal crisis. Therefore, the decision to treat empirically with intravenous methylprednisolone required careful consideration of the balance between the risk of untreated GCA, including irreversible ischaemic complications, and the potential adverse effects of steroid therapy in systemic sclerosis.

Ultimately, the presence of jaw claudication, progressive tongue necrosis, elevated inflammatory markers, and bilateral positive temporal artery ultrasound halo signs provided sufficient concern for GCA to justify treatment, despite the diagnostic uncertainty and competing explanations related to systemic sclerosis-associated vasculopathy.

**Key learning points:**

• Common is common if the patient has suggestive features of GCA, we need to treat them with high-dose steroids to avoid further complications.

• Scleroderma is a disease with widespread vascular manifestations and organ involvement.

• Early treatment can prevent severe complications in GCA.

